# Supplementary material for: The Physiological Mechanism of Exogenous Melatonin on Improving Seed Germination and the Seedling Growth of Red Clover (Trifolium pretense L.) under Salt Stress
Source: Plants (Basel). 2024 Sep 8;13(17):2527. doi: 10.3390/plants13172527 (PMC11397702; doi:10.3390/plants13172527)
Supplement: Supplementary file 1 [file plants-13-02527-s001.zip › plants-3184332-supplementary.pdf]

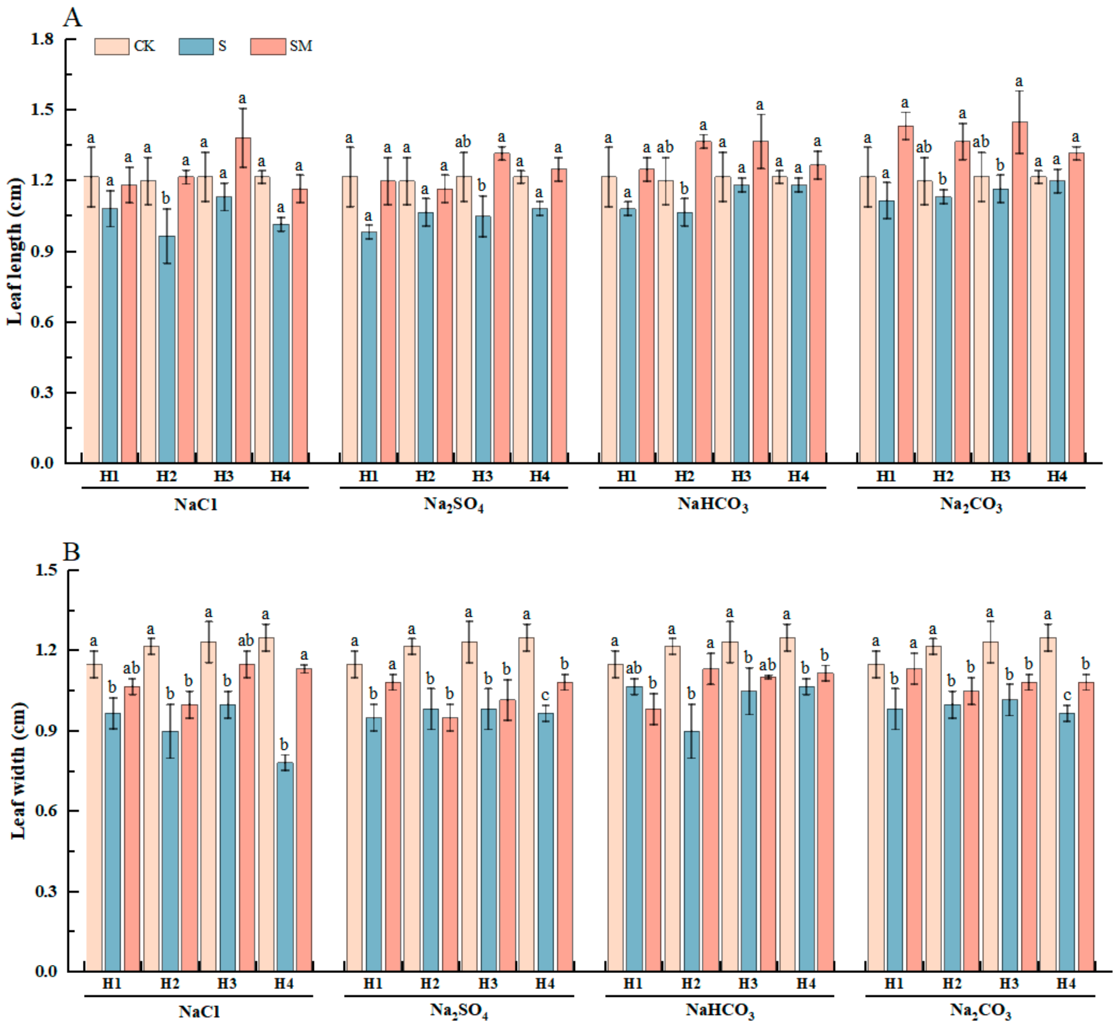

Figure S1. Effect of exogenous MT on photosynthetic characteristics of red clover seedlings under different types of salt stress. (**A**) Changes in leaf length under different treatments; (**B**) Changes in leaf width under different treatments. The error bar in the figure represents the standard deviation (SD, n=3). Different letters indicate significant differences by Tukey's test ($p < 0.05$).

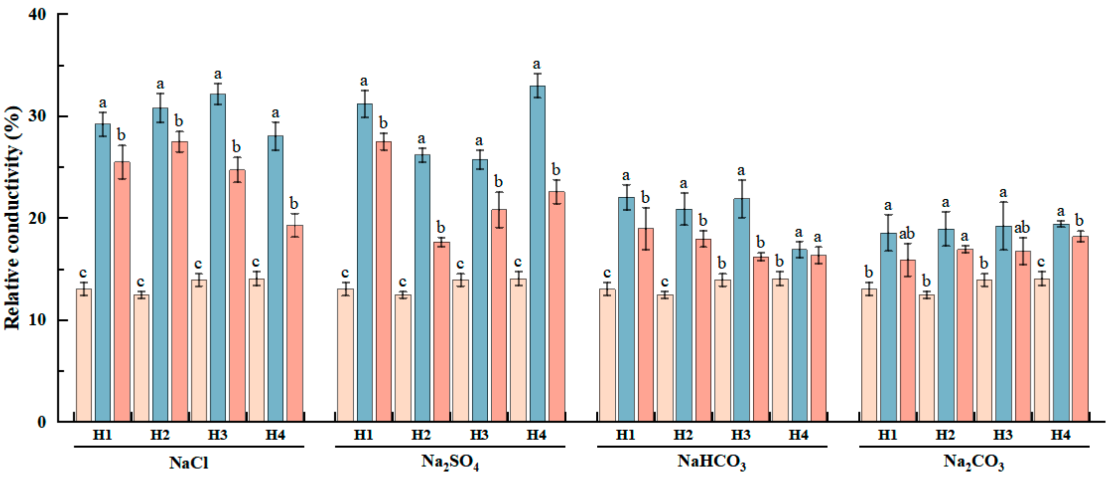

Figure S2. Effect of exogenous MT on the relative conductivity of red clover seedlings under different salt stresses. The error bar in the figure represents the standard deviation (SD, n=3). Different letters indicate significant differences by Tukey's test($p < 0.05$).

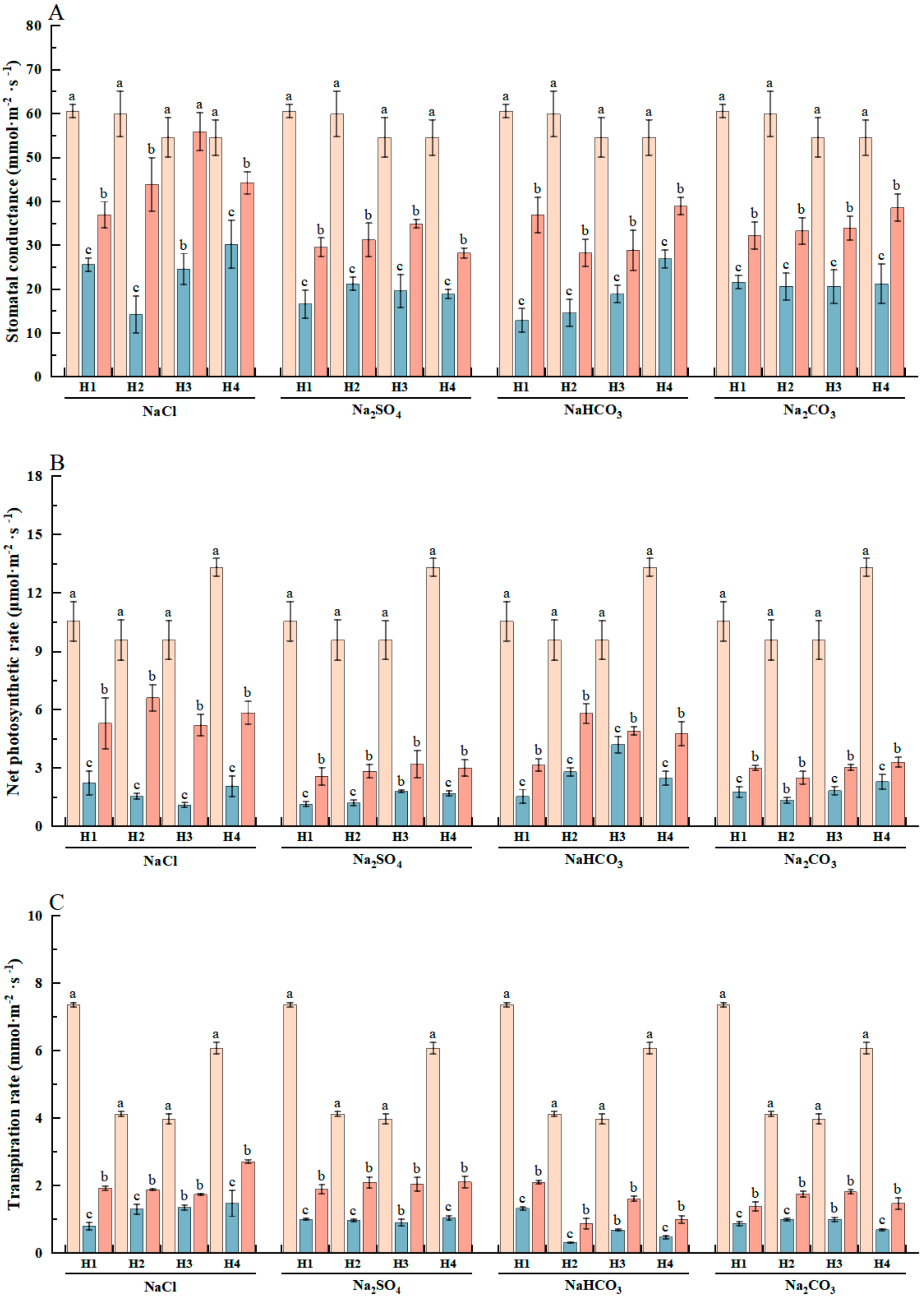

Figure S3. Effect of exogenous MT on photosynthetic characteristics of red clover seedlings under different types of salt stress. (**A**) Changes in net photosynthetic rate under different treatments; (**B**) changes in stomatal conductance under different treatments; (**C**) changes in transpiration rate under different treatments. The error bar in the figure represents the standard deviation (SD, n=3). Different letters indicate significant differences by Tukey's test($p < 0.05$).
